# Chemical analysis of toxic elements: total cadmium, lead, mercury, arsenic and inorganic arsenic in local and imported rice consumed in the Kingdom of Saudi Arabia

**DOI:** 10.1007/s10653-024-02280-0

**Published:** 2024-11-16

**Authors:** May M. Alrashdi, Abby Ragazzon-Smith, Ilya Strashnov, David A. Polya

**Affiliations:** 1 Department of Chemistry, School of Natural Sciences, Faculty of Science and Engineering, The University of Manchester, Manchester, M13 9PL UK; 2 Department of Earth and Environmental Sciences, School of Natural Sciences, Faculty of Science and Engineering, The University of Manchester, Manchester, M13 9PL UK; 3 Chemistry Department, College of Science, Jouf University, P.O. Box: 2014, Sakaka, Kingdom of Saudi Arabia

**Keywords:** Rice, Toxic elements, Hassawi rice, Kingdom of Saudi Arabia, Inorganic arsenic, Good health and well-being

## Abstract

Rice consumption is a pathway for human exposure to toxic elements. Although rice is a major staple in the Kingdom of Saudi Arabia (KSA) there is limited published data about its toxic element composition. Both imported and locally grown Hassawi rice in Saudi Arabia were collected, digested then analysed by HPLC-ICP-MS for inorganic arsenic (i-As) and by ICP-MS for As, Cd, Pb and Hg. Of these toxic elements, i-As was present at concentrations that might give rise to material concerns about human exposure and public health. Hassawi rice (mean 43 ± 5 µg/kg) was found to have significantly lower concentrations of i-As than imported rice (mean 73 ± 8 µg/kg). The estimated exposure of adults consuming imported rice in one KSA city reached 0.3 µg/kg-bw/day, within the margin of safety of the recently withdrawn WHO PTWI for i-As of 2.1 µg/kg-bw/day and higher than EFSA’s 0.06 µg/kg-bw/day skin cancer BMDL_05_.

**Supplementary Information:**

The online version contains supplementary material available at 10.1007/s10653-024-02280-0.

## Introduction

Toxic element, notably heavy metal and metalloid, contamination of food has become a major concern in many parts of the world because these toxic elements may accumulate in biological systems through exposure to contaminated water and soil sources (Banerjee et al., 2013; FDA, 2016). Toxic elements are chemical elements that occur naturally in the Earth's crust and can be concentrated through environmental pollution or industrial and human activities and have harmful effects on humans (FDA, 2016; IARC, 2019). Toxic elements such as arsenic (As), cadmium (Cd), lead (Pb), and mercury (Hg) have been classified as the most toxic substances by the Agency for Toxic Substances and Disease Registry (ATSDR, 2022). Exposure to toxic elements such as inorganic arsenic (i-As), Cd and Pb can lead to human cancers impacting several organs (Xu & Polya, 2021) including the skin, liver, bladder, kidneys, and lungs (FDA, 2016; IARC, 2019). Further, mercury (Hg) has the potential to harm the central nervous system (Park & Zheng, 2012). Therefore, the Food and Agricultural Organization (FAO)/World Health Organization (WHO) Joint Expert Committee on Food Additives (JECFA) has established provisional tolerable weekly intakes (PTWI) for all of As, Cd, Pb and Hg (WHO, 1972, 2013) to reduce the potential health risks of toxic element exposure from food. Rice is a staple food for more than half of the world's population for both economic and agricultural reasons. It is a good source of carbohydrate, vitamins and protein (Stone, 2008). In the Kingdom of Saudi Arabia (KSA), rice occupies a large part of the daily diet, with a mean consumption of more than 47 kg per capita annually (Alamri et al., 2020). The local production of rice in the Kingdom of Saudi Arabia is limited; Hassawi rice is the type that has adapted to the climate of eastern Saudi Arabia. There are two types of Hassawi rice: Hassawi-1 (first degree), the wild type and the other cultivar Hassawi-2 which is a dwarf hybrid of Hassawi-1 (Zhang et al., 2012). Due to climate challenges and the relatively low rates of rice production compared to the rates of rice consumption of the KSA population (Alotaibi et al., 2023), the Kingdom of Saudi Arabia resorts to rice imports from abroad to meet this relatively large consumption, mainly from India (SFDA, 2022). It is estimated that in 2019/21 alone the Kingdom of Saudi Arabia imported 1325 kt/year of rice (OECD, 2022).

Because data on toxic elements and inorganic arsenic in local Saudi rice is relatively sparse, the objective of this study was to measure and compare toxic elements contents in locally grown and imported rice in the Kingdom of Saudi Arabia and use these new data to assess possible risks for human health.

## Previous studies of toxic elements concentrations (As, i-As, Cd, Pb and Hg in the rice imported into the Kingdom of Saudi Arabia

There are a limited number of published studies available in Web of Science on the composition of toxic elements in rice imported into the Kingdom of Saudi Arabia, these are summarised in Table 1. Further, at the time of writing, there was no published study on arsenic speciation in locally grown (Hassawi) rice. Relevant studies were found by using the search string “SAUDI ARABIA” AND “RICE” AND (“TRACE ELEMENTS” OR “TOXIC ELEMENTS” ARSENIC OR “INORGANIC ARSENIC” OR LEAD OR MERCURY OR CADMIUM). Thus, only papers which reported the concentration of any of total arsenic (T-As), inorganic arsenic (i-As), cadmium (Cd), lead (Pb) or mercury (Hg) concentration in rice consumed in the Kingdom of Saudi Arabia (KSA) were included. It is noted that the included articles reported compositions of rice imported from all of India, USA, Australia, Pakistan and Thailand (Alamri et al., 2020; USDA, 2022).

**Table 1:** Previous studies of mean concentrations of As, i-As, Cd, Pb and Hg in rice imported into the Kingdom of Saudi Arabia

Analyte	n (number of samples)	Mean concentration (µg/kg) ± SD	Country of origin	References
T-As	17	16 ± 1	Pakistan	(Ashraf & Alanezi, 2024)
	20	18 ± 1	Thailand	(Ashraf & Alanezi, 2024)
	483	100 ± 24	India	(Almutairi et al., 2021)
	5	49 ± 4.9	Saudi Arabia	(Althobiti et al., 2018)
	8	30	USA and India	(Mohamed et al., 2017)
	6	200 ± 25	Thailand	(Shraim, 2017)
	9	147 ± 55	Pakistan	(Shraim, 2017)
	38	103 ± 48	India	(Shraim, 2017)
	1	188	Australia	(Shraim, 2017)
	10	72 ± 25	India	(Shraim, 2012)
	1	106	Australia	(Shraim, 2012)
	1	63	Pakistan	(Shraim, 2012)
	5	102 ± 14	Thailand	(Shraim, 2012)
	6	132 ± 52	USA	(Shraim, 2012)
i-As	483	38 ± 15	India	(Almutairi et al., 2021)
Cd	17	4.6 ± 0.3	Pakistan	(Ashraf & Alanezi, 2024)
	20	5.8 ± 0.2	Thailand	(Ashraf & Alanezi, 2024)
	483	19 ± 5	India	(Almutairi et al., 2021)
	8	30	USA and India	(Mohamed et al., 2017)
	8	11 ± 6	USA	(Shraim, 2017)
	6	12 ± 5	Thailand	(Shraim, 2017)
	8	25 ± 14	Pakistan	(Shraim, 2017)
	37	18 ± 9	India	(Shraim, 2017)
	17	27.5 ± 39.9	India	(Al-Saleh & Shinwari, 2001)
	4	13.4 ± 3.5	Thailand	(Al-Saleh & Shinwari, 2001)
	2	6.2	USA	(Al-Saleh & Shinwari, 2001)
	2	6.3	Australia	(Al-Saleh & Shinwari, 2001)
Pb	17	14 ± 1	Pakistan	(Ashraf & Alanezi, 2024)
	20	17 ± 2	Thailand	(Ashraf & Alanezi, 2024)
	483	23 ± 8	India	(Almutairi et al., 2021)
	8	40	USA and India	(Mohamed et al., 2017)
	22	31 ± 44	India	(Shraim, 2017)
	4	19 ± 7	Thailand	(Shraim, 2017)
	4	20 ± 2	Pakistan	(Shraim, 2017)
	6	41 ± 30	USA	(Shraim, 2017)
	17	173 ± 356	India	(Al-Saleh & Shinwari, 2001)
	4	37.8 ± 20.4	Thailand	(Al-Saleh & Shinwari, 2001)
	2	88.5	USA	(Al-Saleh & Shinwari, 2001)
Hg	17	36 ± 20	Pakistan	(Ashraf & Alanezi, 2024)
	20	90 ± 170	Thailand	(Ashraf & Alanezi, 2024)
	483	2 ± 0.4	India	(Almutairi et al., 2021)
	17	1.6 ± 1.0	India	(Al-Saleh & Shinwari, 2001)
	4	1.8 ± 1.8	Thailand	(Al-Saleh & Shinwari, 2001)
	2	23.7	USA	(Al-Saleh & Shinwari, 2001)

## Materials and methods

### Data & data quality objectives (DQOs)

Analytical requirements were for the determination of the concentrations T-As, i-As, Cd, Pb and Hg in rice samples with accuracy and precision of better than ± 10% at 3 × detection limit (DL) and a DL of better than 0.01 µg/L in the digested samples, corresponding to a DL of approximately 1 µg/kg for T-As, Cd, Pb and Hg and 0.5 µg/kg for i-As in rice with the sample dilutions utilised in this study.

### Samples analysed in this study

Rice samples (n = 24) were collected from Riyadh markets based on (i) local consumer preferences (Alamri et al., 2020; Al-Saleh & Abduljabbar, 2017; USDA, 2022) (ii) the types of rice that constitute the majority of the rice market in the Kingdom of Saudi Arabia (long-grain rice 90%, medium-grain rice 10%) (USDA, 2022) and (iii) the countries from which Saudi Arabia imports the most rice, viz. India, USA, Australia, Pakistan and Thailand, representing about 99% of the total imported rice Saudi market (Alamri et al., 2020; USDA, 2023).

Locally grown rice (Hassawi rice) was obtained from the rice farms and market of Al-Qurain village in the Al-Ahsa governorate in the eastern province of KSA (n = 9), where rice is produced in KSA due to the region’s suitable climate and availability of water resources. Its production is not enough to meet the broader demand for rice within the country. Thus, Hassawi rice constitutes a small percentage of the locally grown rice market in KSA.

### Sample preparation and analysis

#### Chemicals and standards

Analytical reagent-grade nitric acid (70% wt/wt HNO_3_) (Fisher Scientific (AR)) was used for sample and standard preparation. The nitric acid was further purified in the laboratory using sub-boiling acid distillation (DST-1000 acid purification system, Savillex). The purity of the acid and water was regularly checked and confirmed by ICPMS measurements to be <  < 1 µg/L for most the target analytes As, Cd, Pb and Hg. Arsenic (As), arsenite (As(III)), arsenate (As(V)) (Sigma-Aldrich, 1000 µg/mL), dimethylarsinic acid (DMA) disodium salt tri hydrate (Merck Life Science), monosodium acid methane arsonate sesquihydrate (MSMA) (CHEM Service), cadmium (Cd), lead (Pb) (VWR Avantor, 1000 µg/mL), (Sigma-Aldrich, 1000 µg/mL) and mercury (Hg) (Sigma-Aldrich, 10,000 µg/mL) standards for ICP were used for calibration standard preparation. For all sample preparation, a water purification system (Avidity Science) was used to provide 18.2 MΩ type 1 ultrapure laboratory water.

#### Calibration and internal standards

The preparation of all the calibration standard solutions was carried out by serial dilution of the stock solutions (FDA method EAM 4.11 and 4.7) (Gray et al., 2020; Kubachka et al., 2012).

For total As, Cd and Pb measurement using ICP-MS, eight mixed calibration standards with concentrations 0, 0.05, 0.1, 0.2, 0.5, 1, 1.5 and 2 µg/L were used. While for ICP-MS Hg only calibration standards with the same concentrations from 0 to 2 µg/L was used.

For arsenic speciation, separate calibration standards were prepared for each As species arsenite (As(III)), arsenate (As(V)), monomethyl arsonic acid (MMA) and dimethylarsinic acid (DMA). Seven calibration standards with concentrations of 0, 0.1, 0.2, 1, 2, 10 and 20 µg/L were used to bracket the range of concentrations anticipated in the rice digests.

A full set of calibration standards were analysed as unknowns at both the beginning and the end of each analytical run, as well as after every fifteen samples. A weighted calibration curve using Eqs. (1), (2) and (3) from (Miller & Miller, 2018) was employed to account for and recognising that analytical imprecision is heteroscedastic i.e. it varies with concentration.

1 $$W_{i} = \frac{{s_{i}^{ - 2} }}{{\mathop \sum \nolimits_{i} s_{i}^{ - 2} /n}}$$

2 $$S_{{\hat{x}0w}} = \frac{{S_{{\left( {y/x} \right)w}} }}{b}\sqrt {\frac{1}{n} + \frac{1}{{w_{i} }} + \frac{{(y_{0} - \overline{y})^{2} }}{{b^{2} \mathop \sum \nolimits_{i = 1}^{n} (w_{i} xi - n\overline{x}w)^{2} }}}$$

3 $${S}_{(y/x)w}=\sqrt{\frac{\sum_{i}{w}_{i}({y}_{i}-{\widehat{{y}_{i}})}^{2}}{n-2}}$$

where **W**_**i**_ is the weighting of the i-th calibration standard,** s**_**i**_ is the standard deviation of the signal response for the i-th calibration standard; **n** is the number of calibration standards**;** ${S}_{\widehat{x}0w}$ is the weighted standard deviation of the estimated concentration of the sample; **S**_**(y/x)w**_ is a weighted standard deviation defined by Eq. (3); **y**_**0**_ is the measured signal for the sample; $\overline{\user2{x}}$ is the mean concentration of the calibration standards, $\overline{{\varvec{y}} }$ is the mean signal of the calibration standards, **b** is the slope of the weighted first order linear regression curve for the calibration standards and $\widehat{{{\varvec{y}}}_{{\varvec{i}}}}$ is expected value of y_i_ according the weighted regression curve.

Any possible contamination arising from sample preparation was estimated from procedural blanks measured at regular intervals. Separately, analytical blank samples were analysed to check the purity of the reagents to check for any contamination arising, including from sample containers. A standard solution of Rh (10 µg/L, Alfa Aesar) was introduced into the ICP parallel to the sample solution and used as an internal standard to adjust for variations in signal sensitivity. Only for HPLC-ICP-MS measurements of arsenic speciation, the internal standard solution was also mixed with a 10% hydrogen peroxide solution to aid in ionisation of As(III) and to improve chromatographic separation of inorganic arsenic from DMA.

#### Standard reference materials (SRMs)

Well characterised (for total As, i-As, Cd, Pb and Hg content) (NIST, 2021) rice flour samples were purchased from the National Institute of Standards and Technology (NIST, sample 1568b) and used for quality control. In addition, the robustness of our procedures were successfully tested and confirmed through our participation in an inter-laboratory comparison exercise organised and run by the UK Laboratory of the Government Chemist (LGC).

#### Pre-treatment of rice samples

Rice samples were ground using a mortar and pestle and stored in a plastic bag at room temperature until digested.

#### Sample preparation

For sample preparation, the methods published by FDA were adopted for total arsenic (The Elements Analysis Manual (EAM), Sect. 4.7 (Gray et al., 2020) and for arsenic speciation ((The Elements Analysis Manual (EAM), Sect. 4.11 (Kubachka et al., 2012)). For total arsenic analysis by ICP-MS, ground rice samples (0.5 g) including certified reference materials (CRM) were digested in triplicate in 8.0 mL sub-distilled concentrated nitric acid (69–70% wt/wt) and 1 mL of high purity 30% wt/wt hydrogen peroxide (Merck Life Science) using closed-vessel style microwave digestion tubes (MARSXpress) followed by microwave digestion using a Mars (240/50) Microwave System(CEM). All samples were diluted up to 50 ml with deionised water followed by 5 mL of 10% v/v hydrochloric acid (AR grade, Fisher Scientific) and further deionised water to a final volume of 100 mL. Samples were filtered using a 0.2 µm polypropylene filter (Fisher Scientific) and 10 mL syringes (IVS10, HMC premedical S.P.A). Then 5 mL of the filtered sample was topped up with 6 mL of deionised water, providing samples with a nitric acid concentration (2%) required for ICP-MS analysis whilst at the same time bringing total expected concentrations into the optimal range for instrumental sensitivity (Gray et al., 2020).

For arsenic speciation, a softer digestion approach was used in order to ensure preservation of the species of interest. Ground rice (1.0 g) was crushed and mixed with 10 mL of 0.28 M HNO_3_ in the polypropylene vials. The mixture was heated in a block digestion system (Anton Paar) at 95 °C for 90 min, and then diluted with 6 mL of 18 MΩ deionised water. Each sample was centrifuged at 3000 rpm for 10 min and filtered through a 0.2 µm polypropylene filter / 10 mL syringe. Then 0.5 mL of the filtrate was mixed in an HPLC vial (Thermos Scientific 1.5 mL) with 1 mL ammonium hydroxide (28.0%—30.0%, ACS Reagent, Sigma-Aldrich) solution to provide a solution with pH of 9.95 ± 0.05. The stability of arsenic speciation in digests was evident from the repeated analysis of selected samples and also inferred from previous studies (Mondal et al., 2010; Polya & Watts, 2017; Polya et al., 2003).

#### Analytical instrumentation

A CEM Mars (240/50) Microwave System was used to prepare samples for total toxic elements analysis. It operates using microwave energy to generate heat in the samples, resulting in rapid heating and increased pressures, hence facilitating expedited and efficient digestion of the samples. MARSXpress vessels were used to contain the samples and reagents during the digestion process with up to 40 samples and/or standards digested at a time.

For arsenic speciation analysis, acid digestion was performed in a hot block digestion system (Anton Paar) equipped with up to 48 sample vials (50 mL), capable of heating up to 360°C, the error on temperature measurements is 1°C.

**Fig. 1:**
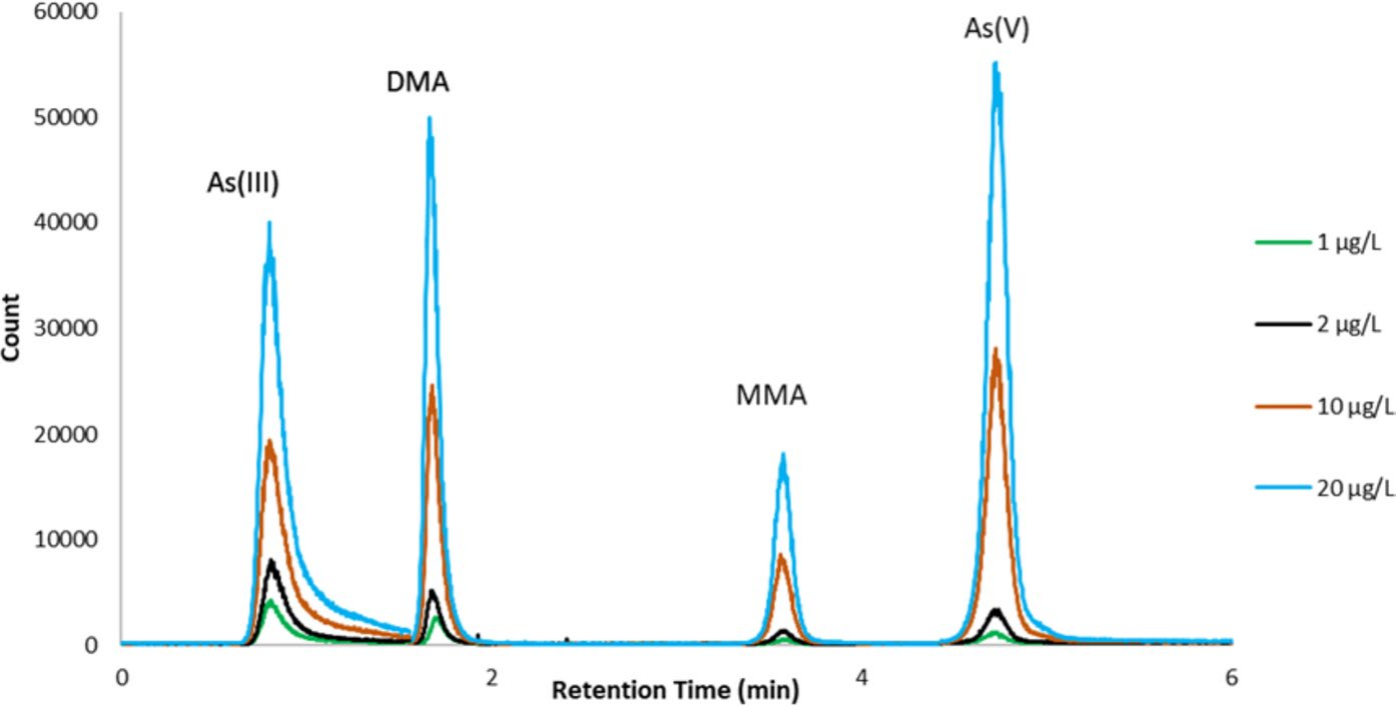
A typical ion chromatogram for arsenic speciation (1, 2, 10 and 20 µg/L) showing a good separation of As(III), DMA, MMA and As(V) using the PRP-X 110S anion exchange column. Analysis by HPLC-ICP-MS (Agilent 1260 chromatography system coupled with an Agilent 7700 ICP-MS)

The microwave and heating block system were programmed for power and temperature as a function of, time following the relevant FDA methods (Gray et al., 2020; Kubachka et al., 2012) as illustrated in Table S1. Total As, Cd and Pb were determined by ICP-MS (Inductively Coupled Plasma Mass Spectrometry) Agilent 8900 Triple Quadrupole. Total Hg was determined by ICP-MS Agilent 7700. Arsenic speciation, including inorganic arsenic (i-As), was determined by High Performance Liquid Chromatography coupled with an Inductive Coupled Plasma Mass Spectrometer (HPLC-ICP-MS) (Agilent 1260; Agilent 7700). The instrument conditions of ICP-MS and ICP-MS/MS are listed in Tables S2 and S3, the elution order of arsenic species (As(III). DMA, MMA, and As(V)) are shown in Fig. 1.

For pipetting, ultra-high precision electronic pipettes were used: Sartorius Picus ^R^ NxT and Sartorius Proline Plus 300 µL, 1 mL, 5 mL having systematic errors of better than 0.1–1%, depending on the sampling volume, calibrated according to ISO 8655:2022.

### Assessment of toxic elements daily intake (EDI)

The estimated daily intake (human exposure to a heavy metal/metalloid contaminants), from consumption of rice was estimated by Eq. (4) developed by the United States Environmental Protection Agency (USEPA, 1992):

4 $${\mathbf{EDIi}}{ } = C_{i} \times \left( {\frac{{ IR_{r} }}{bw}} \right)$$

Where.

EDI_i_ = the estimated daily intake of the subscripted contaminant,

C_i_ = the concentration of the subscripted contaminant in raw rice (dry weight basis),

*IR*_r_ = daily intake rate of raw rice (dry weight basis),

bw = body weight.

In particular, because there was no access to the water in which different individuals cooked their rice, we ignored the contribution of cooking water, on average at a population scale, for the purposes of the exposure calculations. This appears to be a reasonable first-order approximation in agreement with (Mondal et al., 2010) who noted that cooking water contributed relatively little to inorganic arsenic exposure compared to inorganic exposure from raw rice.

The mean intake of rice by adults in the Kingdom of Saudi Arabia was taken to be 0.128 kg/person/day which was the average rice consumption reported recently (see Table 2). We also separately considered a figure for a sub-population from the city of Najran, viz. 0.243 ± 0.086 kg/person/day for both males and females over 18 years old—this figure is taken from a detailed survey and statistical evaluation of the mean daily rice intake by (Mohamed et al., 2017). This datum is somewhat higher (see Table 2) than other estimates of per capita rice consumption for the whole of the Kingdom of Saudi Arabia, consistent with higher rates of rice consumption in large cities within the Kingdom. For Saudi children of both genders, there is a lack of available data of the rate of rice consumption, so for this group of the population we derived rice consumption from the carbohydrate intake per day estimated by (Kutbi, 2021) noting that the typical carbohydrate content of rice is approximately 80% of its total dry weight (USDA, 2019b). The relative contribute of rice to the total carbohydrate intake reported by (Kutbi, 2021) was determined by combining the carbohydrate consumption rates for children reported by (OECD, 2022) for rice, wheat, maize and coarse grain for the Kingdom of Saudi Arabia with previous estimates of the carbohydrate contents of those major carbohydrates sources, viz. rice 79%, wheat,71% maize 74% and coarse grain 74% (USDA, 2019a, 2019b, 2020, 2021).

**Table 2:** Summary of daily per capita rice consumption rates in the Kingdom of Saudi Arabia derived from published values for mean per capita carbohydrate intake per day for children and mean per capita rice consumption rates in the Kingdom of Saudi Arabia determined for adults

Population group	Reported parameter and unit	Derived daily per capita rice consumption kg/person/day	References
Children: males 6–8 years	162 g carbohydrate/day	0.018	(Kutbi, 2021)
Children: females 6–8 years	163 g carbohydrate /day	0.018	
Children: males 9–12 years	170 g carbohydrate /day	0.018	
Children: females 9–12 years	153 g carbohydrate /day	0.017	
Adult’s males and females; > 18 years	1244 kt consumed rice/year	0.133	(OECD, 2022)
	47 kg rice/person/year	0.129	(Alamri et al., 2020)
	40 kg rice/person/year	0.110	(Almutairi et al., 2021)
	51 kg rice/person/year	0.140	(FAOSTAT, 2022)
	243 g rice/person/day	0.243	(Mohamed et al., 2017)

The proportion of population of various ages and genders and accordingly the distribution of body weight distribution according to the General Authority for Statistics Kingdom of Saudi Arabia (GASTAT, 2021) and (Al Othaimeen et al., 2007) respectively were used to calculate the weighted mean body weight of both children and adults for exposure and risk estimation as summarized in Table 3.

**Table 3:** Distribution of age and gender in KSA and age and gender dependent body weights based on (GASTAT, 2021), in turn based in part on (Al Othaimeen et al., 2007). SD refers to standard deviation

Age	Proportion of population		Males		Females	
	Males	Females	wt (kg)	SD (kg)	wt (kg)	SD (kg)
< 4	0.04	0.04	13.23	4.92	14.12	6.10
5–9	0.04	0.04	22.96	8.30	22.98	9.80
10–14	0.04	0.04	39.85	11.50	41.98	11.50
15–19	0.04	0.03	58.27	14.80	54.15	16.20
20–24	0.04	0.04	62.42	14.30	55.80	13.70
25–34	0.10	0.08	67.78	13.80	61.44	16.50
35–44	0.13	0.08	71.66	13.90	66.40	17.70
45–54	0.08	0.04	69.61	13.70	65.35	15.40
55–64	0.04	0.02	67.30	14.10	62.22	15.20
65 and above	0.02	0.02	65.67	15.80	60.56	24.00

### Assessment of lifetime carcinogenic risks

Lifetime carcinogenic risks attributable to exposure to trace elements in rice by consumers in KSA were calculate according to a (USEPA, 1989) one-hit model using Eq. (5):

5 $${\text{CR}}_{{\text{i}}} \, = \,{\text{CPS}}_{{0,{\text{i}}}} \, \times \,{\text{EDI}}_{{\text{i}}}$$

where CR_i_ is the lifetime excess carcinogenic risk attributable to exposure to the subscripted toxic element, i; CPS_0,i_ is the cancer slope factor for the subscripted toxic element, i and EDI_i_ is the estimate daily exposure to the toxic element, i as calculated by Eq. (4). Values used for cancer slope factors were as follows: As (1.5 × 10^−3^ (µg/kg-bw/day)^−1^), Cd (3.8 × 10^−4^ (µg/kg-bw/day) ^−1^), and (Pb 8.5 × 10 ^6^ (µg/kg-bw/day)^−1^) (Masri et al., 2021; Real et al., 2017; USEPA, 1991). No published CSF_0_ values for Hg were found, presumably as its health effects are largely non-carcinogenic, notably neurotoxicity (USEPA, 1995) and so CR_i_ values for Hg were not estimated.

### Probability distributions and statistical test for input variables

Different probability distributions of i-As, Cd, Pb, and Hg concentration in imported and local rice were established using Origin 2022. It was performed by selecting the data and running the test via the distribution’s fit menu. It was run for multiple distributions available, including normal, lognormal, Weibull, exponential and gamma. Then the best fitted distribution were selected using Akaike information criterion (AIC) calculated using (Eq. 6). AIC values were compared for different fitted distributions, and the distribution with the lowest AIC selected as  the best fit (Table S4). These distributions are presented for illustrative purposes and theoretically could be used to perform Monte Carlo modelling to work out the exposure distribution. However, in this study the exposures were calculated using series values ​​for different population groups namely Saudi adults, Saudi city adults and Saudi children.

6 $$AIC=2k-2\text{ln}(L)$$

where.

k = number of model parameters.

L = the maximum value of the likelihood function of the model.

The Mann–Whitney U test was used to determine whether there was a significant difference in the mean of toxic elements concentration for imported and local rice because it is a non-parametric test for comparing independent sets of samples not requiring the distributions to be normal, and using the following Eq. (7) (Corder & Foreman, 2011):

7 $${U}_{1}={n}_{1}{n}_{2}+\frac{{n}_{1}({n}_{1}+1)}{2}-{R}_{1} \text{and }{U}_{2}={n}_{1}{n}_{2}+\frac{{n}_{2}({n}_{2}+1)}{2}-{R}_{2}$$

where n_1_ = number of group 1, n_2_ = number of group 2, R_1_ = sum of the ranks for group 1, and R_2_ = sum of the ranks for group 2.

## Results and discussion

### Analytical quality control data

Analytical accuracy was evaluated using (i) NIST 1568b rice flour reference material (SRM) with indicated recoveries of 107%, 104%, 92%, 81% and 113% for T-As, Cd, Pb, Hg and i-As respectively; (ii) a Laboratory of the Government Chemist (LGC) inter-laboratory comparison scheme which resulted a recovery of 112%, 95% of T-As and 84%, 88% of i-As for sample A and B respectively as shown in Table 4. The precision of the analyses was assessed by replicate analyses and found to be typically ± 10% for most elements. The precision for Hg, however, was 19%. The relatively low or variable recoveries for Hg and i-As reflect proximity of the measured concentrations to the respective method detection limits, the volatility and sorption behaviour of Hg, and widely acknowledged challenges to i-As speciation analysis, the latter being reflected in the US FDA’s adoption of 100% ± 30% as an acceptable level of recovery for i-As analyses in food by their methods (FDA, 2022).

**Table 4:** Evaluation of the accuracy of the analytical methodology used to determine T-As, Cd, Pb, Hg and i-As using reference material (SRM) Rice Flower-NIST 1568b and the results of inter-laboratory comparison experiments conducted by external company (LGC)

Analyte	Certified value (µg/kg) ± Standard deviation	Obtained value (µg/kg) ± Standard deviation	Recovery	SRM
T-As	285 ± 7	304 ± 30	107% ± 11%	NIST, sample 1568b
Cd	22.4 ± 1.3	23 ± 1	103% ± 7%	
Pb	8 ± 3	7 ± 1	92% ± 37%	
Hg	5.91 ± 0.36	5 ± 2	85% ± 34%	
i-As	92 ± 10	104 ± 12	113% ± 18%	
T-As	265 ± 5	296 ± 18	112% ± 7%	LGC sample A
i-As	108 ± 3	91 ± 17	84% ± 16%	
T-As	132 ± 3	125 ± 7	95% ± 5%	LGC sample B
i-As	82 ± 3	72 ± 22	88% ± 27%	

Expressed as % recoveries and standard deviation SD respectively, n = 3

### T-As, i-As, Cd, Pb and Hg concentration in rice

There was a higher concentration of inorganic arsenic in imported rice than in locally grown rice (Hassawi rice) commonly consumed in the Kingdom of Saudi Arabia, as shown by the data in Table 5 and the following text. Table 5 shows the measurement result for the total of As, Cd, Pb, and Hg in individual samples of both imported and local rice in KSA.

**Table 5:** Measured concentration of toxic elements (T-As, Cd, Pb and Hg) and i-As ± standard deviation ((SD) n = 3) (µg/kg) in local and imported rice consumed in the Kingdom of Saudi Arabia

No	Origin	Rice type	T-As (µg/kg) ± SD	i-As (µg/kg) ± SD	Cd (µg/kg) ± SD	Pb (µg/kg) ± SD	Hg (µg/kg) ± SD
*Imported rice samples*							
1	India	Yellow long grain	93 ± 8	61 ± 7	25 ± 4	109 ± 3	7 ± 3
2	India	Yellow long grain	55 ± 8	55 ± 6	14 ± 1	29 ± 3	2 ± 3
3	India	Yellow long grain	94 ± 8	63 ± 6	21 ± 1	78 ± 3	2 ± 3
4	India	White long grain	119 ± 8	68 ± 8	22 ± 1	39 ± 3	2 ± 3
5	India	White long grain	72 ± 8	60 ± 7	19 ± 1	33 ± 3	2 ± 3
6	India	White long grain	78 ± 8	65 ± 6	24 ± 1	6 ± 3	2 ± 3
7	India	White long grain	79 ± 8	58 ± 6	24 ± 1	12 ± 3	1 ± 3
8	India	Yellow long grain	98 ± 8	65 ± 5	19 ± 1	64 ± 3	2 ± 3
9	India	Yellow long grain	139 ± 8	88 ± 10	93 ± 4	19 ± 3	2 ± 3
10	India	Yellow long grain	128 ± 8	86 ± 10	18 ± 1	46 ± 3	1 ± 3
11	India	White long grain	87 ± 8	65 ± 8	17 ± 1	7 ± 3	7 ± 3
12	India	Brown long grain	150 ± 8	85 ± 10	22 ± 4	14 ± 3	3 ± 3
13	India	White long grain	133 ± 9	69 ± 8	25 ± 4	14 ± 3	1 ± 3
14	India	Yellow long grain	185 ± 8	91 ± 11	19 ± 1	62 ± 3	1 ± 3
15	India	Yellow long grain	94 ± 9	60 ± 7	14 ± 1	35 ± 3	1 ± 3
16	India	Yellow long grain	149 ± 9	102 ± 12	8 ± 1	4 ± 3	1 ± 3
17	India	Yellow long grain	216 ± 8	100 ± 12	23 ± 1	8 ± 2	11 ± 3
18	USA	Whit medium grain	141 ± 4	52 ± 6	14 ± 1	48 ± 3	3 ± 3
19	USA	Yellow medium grain	223 ± 4	61 ± 7	15 ± 1	79 ± 1	3 ± 1
20	USA	Yellow medium grain	260 ± 8	120 ± 12	21 ± 1	23 ± 4	5 ± 2
21	USA	Yellow medium grain	269 ± 8	103 ± 12	15 ± 1	9 ± 2	3 ± 3
22	Australia	White short grain	144 ± 8	63 ± 7	30 ± 1	13 ± 3	19 ± 3
23	Pakistan	White long grain	110 ± 8	50 ± 6	31 ± 1	18 ± 3	2 ± 3
24	Thailand	White medium grain	182 ± 8	63 ± 7	11 ± 1	7 ± 3	5 ± 3
*Local rice samples (Hassawi rice)*							
25	Al Hasa (Saudi Arabia)	Red medium grain	79 ± 8	43 ± 5	24 ± 4	104 ± 3	3 ± 3
26	Al Hasa (Saudi Arabia)	Red medium grain	75 ± 4	42 ± 5	9 ± 1	47 ± 4	1 ± 3
27	Al Hasa (Saudi Arabia)	Red medium grain	68 ± 8	39 ± 4	9 ± 1	38 ± 3	1 ± 3
28	Al Hasa (Saudi Arabia)	Red medium grain	42 ± 9	41 ± 5	59 ± 4	1 ± 3	0 ± 3
29	Al Hasa (Saudi Arabia)	Red medium grain	51 ± 9	42 ± 5	15 ± 1	1 ± 3	3 ± 3
30	Al Hasa (Saudi Arabia)	Red medium grain	55 ± 9	42 ± 5	99 ± 4	14 ± 3	2 ± 3
31	Al Hasa (Saudi Arabia)	Red medium grain	100 ± 9	49 ± 6	22 ± 4	8 ± 3	3 ± 3
32	Al Hasa (Saudi Arabia)	Red medium grain	50 ± 9	43 ± 5	9 ± 1	26 ± 6	1 ± 3
33	Al Hasa (Saudi Arabia)	Red medium grain	103 ± 6	46 ± 5	23 ± 4	29 ± 8	1 ± 2

**Fig. 2:**
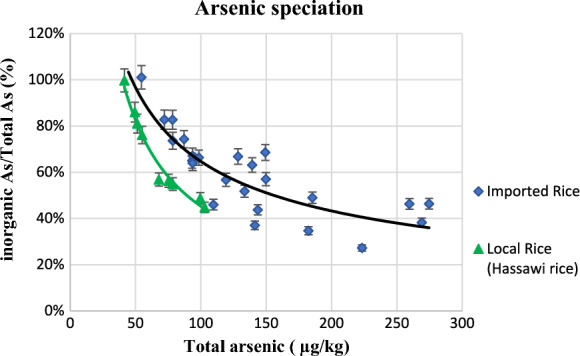
Cross-plot of proportion of arsenic occurring as inorganic arsenic vs total arsenic in samples of imported (blue diamonds) & local rice (Hassawi rice) (green triangles) consumed in the Kingdom of Saudi Arabia

It was found that the content of T-As in the common imported rice consumed in the Kingdom of Saudi Arabia varied from 55 to 223 µg/kg with a mean of 140 µg/kg. However, T-As in local rice (Hassawi rice) ranged from 42 and 103 µg/kg and a mean of 69 µg/kg.

i-As of imported rice was detected in the range between 45 to 120 µg/kg (mean 73 µg/kg) and in Hassawi rice ranged from 39 to 49 µg/kg (mean 43 µg/kg). The mean proportion of arsenic occurring as i-As was 56% and 67% for imported and Hassawi rice respectively as shown in Fig. 2.

In the imported rice the mean concentration of Cd, Pb and Hg was (23 ± 1 µg/kg), (32 ± 3 µg/kg) and (4 ± 3 µg/kg) respectively. While in the local rice, the mean concentration of Cd,Pb and Hg was (30 ± 4 µg/kg), (30 ± 4 µg/kg) and (2 ± 3 µg/kg) respectively.

**Fig. 3:**
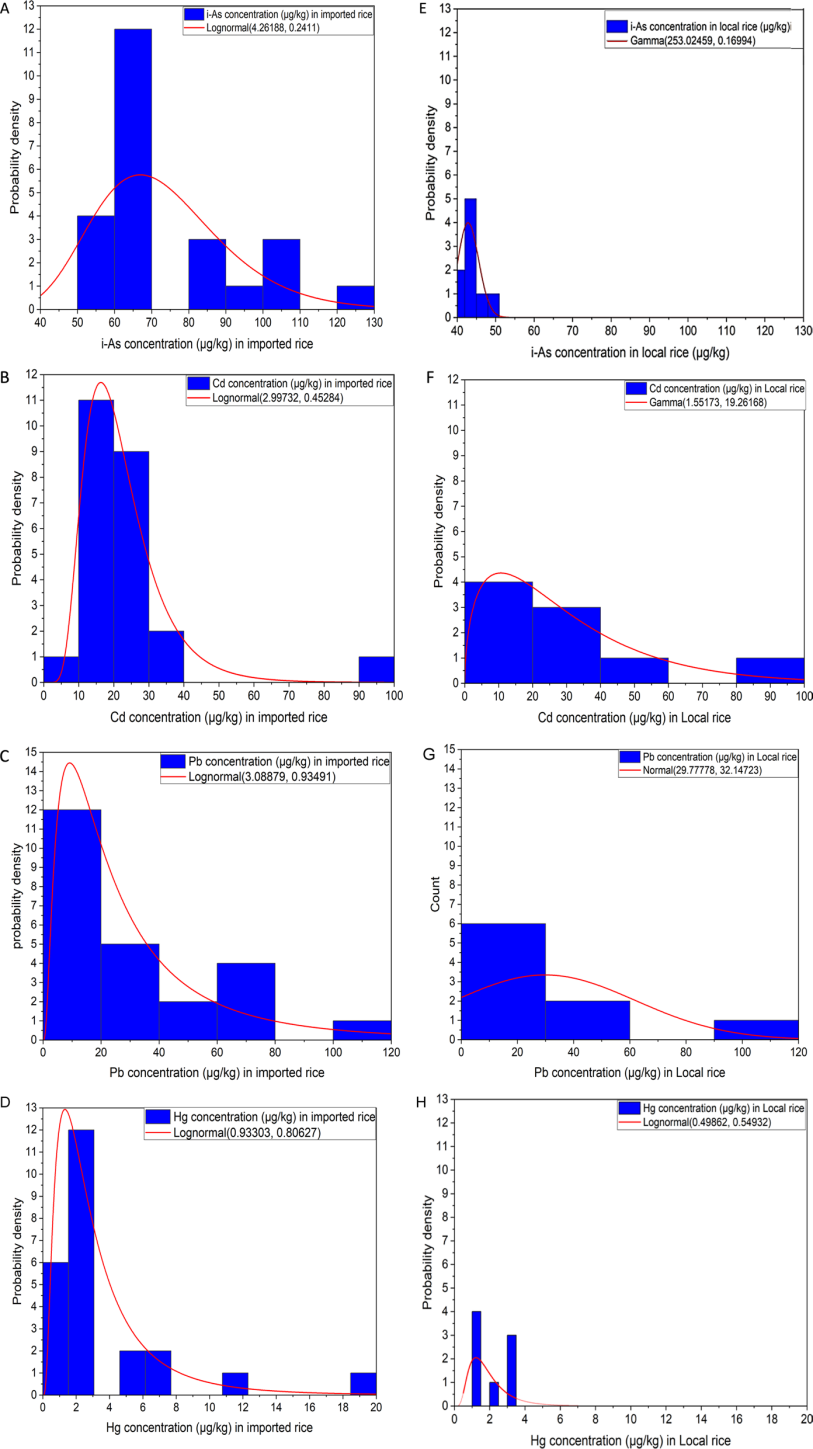
Distributions of the input variables of i-As, Cd, Pb and Cd concentration in imported rice (A-D) and local rice (E–H) (µg/kg) in the Kingdom of Saudi Arabia, established by statistical (Kolmogorov Smirnov test) and graphical (probability plots) methods

Probability distributions for the inorganic As, Cd, Pb and Hg content of imported and local rice are shown in Fig. 3. Log normal distribution was the best fit for i-As concentrations in imported rice and the Gamma function was the best fit for i-As in local rice.

The Mann–Whitney U statistic test (n_1_ = 24, n_2_ = 9, *P* ≤ 0.05 one-tailed) showed that the mean of i-As for imported rice was significantly higher than that for local rice. However, the values of Cd, Pb and Hg of imported rice are not significantly different from local rice at *P* ≤ 0.05 (two-tailed). In addition, U test showed that the means of T-As, Cd and Pb of imported rice (n1 = 24) were not significantly different from previously reported values (Shraim, 2017) (Table 1) (n2 = 63 T-As, 59 Cd, 36 Pb) at *P* ≤ 0.05 (two-tailed). The t-test (n1 = 9, n2 = 5, at P ≤ 0.05 two-tailed) showed that the mean of T-As of local rice was not significantly different from that found by (Althobiti et al., 2018) (Table 1). Furthermore, the t-test showed that the mean of Hg of imported rice (n1 = 24) found in this study was not significantly different from those found in previous studies by (Ashraf & Alanezi, 2024), (Almutairi et al., 2021) and (Al-Saleh & Shinwari, 2001) (Table 1) (n2 = 543 at P ≤ 0.05). However, the mean of T-As, Cd, Pb and i-As of imported rice (n1 = 24) were all significantly different from previously reported values (Ashraf & Alanezi, 2024), (Almutairi et al., 2021), (Shraim, 2012) and (Al-Saleh & Shinwari, 2001) (Table 1) (n2 = 541 T-As, 545 Cd, 526 Pb, i-As 483) at *P* ≤ 0.05 (t-test), which could reflect the representativeness of the samples and differences in the composition of imported rice in different parts of KSA. Both ranges of iAs/As are broadly consistent with the high inorganic arsenic/total arsenic ratios typically found in many rice varieties grown in Asia (Williams et al., 2005).

### Exposure assessment to toxic elements

The typical exposure to toxic elements for 3 different sub-populations in KSA are shown in Table 6. These have been calculated using Eq. (4), using mean measured concentrations of the toxic elements in either imported or locally grown rice and using the daily intake rate of rice and body weight for each sub-population according to Tables 2 and 3. These values may be compared to the Food and Agricultural Organization (FAO) and World Health Organization (WHO) Joint Expert Committee (JECFA) provisional tolerable weekly intake (PTWI) values for inorganic arsenic of 15 µg/kg-bw/week (EFSA, 2009), cadmium (Cd) 7 µg/kg-bw/week, lead (Pb) 25 µg/kg-bw/week and mercury (Hg) 5 µg/kg-bw/week (Jallad, 2015; WHO, 1972, 2013).

**Table 6:** Calculated mean of toxic elements intake based on the weighted mean body weight and rice consumption and male/female ratio in the Kingdom of Saudi Arabia (KSA) and Najran City, KSA

Sub-population & mean body weight	Rice consumption (kg/person /day)	Toxic elements intake from consuming of Imported and (local) rice				
		As intake (µg/kg-bw/day)	i-As intake (µg/kg-bw/day)	Cd intake (µg/kg-bw/day)	Pb intake (µg/kg-bw/day)	Hg intake (µg/kg-bw/day)
Children 39.42 kg	0.018	0.07 (0.03)	0.03 (0.02)	0.01 (0.01)	0.01 (0.01)	0.002 (0.001)
Adults. KSA 66.22 kg	0.128	0.30 (0.13)	0.14 (0.08)	0.04 (0.06)	0.06 (0.06)	0.008 (0.004)
Adults, Najran City 66.22 kg	0.243	0.51 (0.25)	0.27 (0.16)	0.08 (0.11)	0.11 (0.11)	0.01 (0.01)
PTWI	N/A	N/A	2.1	1.0	3.6	0.7

Values not in parentheses refer to imported rice. Values in parentheses refer to local Saudi rice (Hassawi rice)

The results of toxic elements exposure estimation showed that the levels of cadmium, lead and mercury were low compared to the WHO PTWI for both imported and local rice. In contrast, there was concern about the mean concentration of inorganic arsenic in imported rice specifically in rice consumption in the city because this would result to intakes of inorganic arsenic up to 0.3 µg/kg-bw/day which was higher than the benchmark dose lower confidence limit for BMDL_05_ values for skin cancer risks of 0.06 µg/kg-bw/day recently reported by (EFSA et al., 2024) and close to the WHO recently withdrawn PTWI for i-As of 2.1 µg/kg-bw/day (EFSA, 2009). This inorganic arsenic exposure level from imported rice was considered within the margin of exposure (MOE) and margin of safety (MOS) and the ratio of (reference /exposure) is lower than 100 resulting a concern for public health (EFSA, 2023). The estimated excess lifetime carcinogenic risks arising from exposure to toxic elements in rice consumed in KSA were estimated to be (3 × 10^−5^–4 × 10^−4^) from i-As overlapping with or exceeding a widely considered acceptable level of such risks of 1 × 10^−6^–1 × 10^−4^, suggesting that risk mitigation may be necessary. i-As is considered to be a carcinogen and can affect many organs notably the skin, bladder, and lungs (FDA, 2016) whilst it has also been identified as a contributory factor in the development of cardiovascular and other diseases (EFSA et al., 2024). Excess lifetime carcinogenic risks arising from equivalently sourced exposure to Cd was generally lower than for i-As, ranging from 4 × 10^−6^–4 × 10^−5^, whilst the values for Pb were much below/better than widely accepted levels of such risks.

It should be noted that there are a number of factors that may impact on the validity of the conclusions. For example, the bioavailability of toxic elements from rice is an important factor in the estimation of potential health risks. Although the data on the bioavailability of Cd, Pb and As in rice is limited, the relative bioavailability of Cd has been reported to range from 41 to 84% for concentrations 410–1670 µg/kg and for Pb 11 − 59% for 90–880 µg/kg based on kidney and liver (Li et al., 2019; Zhao et al., 2018). As for arsenic, (Juhasz et al., 2006; Laparra et al., 2005) reported that 90% of As is bioavailable in the blood for rice cultivars with higher inorganic content, and which is, in principle, applicable to this study. Further, toxic element exposures might be reduced because of food wastage or because of cooking methods. Lastly, toxic element exposures from rice consumption are critically dependent upon dietary factors, which may vary materially from sub-population to sub-population as highlighted in our work—further research is indicated, therefore, in the distribution of rice consumption rates in sub-populations with the Kingdom of Saudi Arabia.

## Conclusions

The concentrations of the toxic elements As, Cd, Pb and Hg in imported and locally grown rice in the Kingdom of Saudi Arabia have been analysed by ICP-MS and HPLC-ICP-MS. The potential health risks from exposure to toxic elements to children and adults from rice consumption have been evaluated. This study is focused on both local rice (Hassawi rice) and imported rice which are commonly consumed in the Kingdom of Saudi Arabia and constitute 99% of the total Saudi rice market (Alamri et al., 2020). Rice is considered one of the leading foods in KSA, its relatively high consumption levels influenced by culture and traditions. The imported rice (n = 24; mean 73 ± 8 µg/kg) was found to have significantly higher (Mann–Whitney U test (*p* ≤ 0.05; one-tailed) concentrations of i-As than the mean concentration of i-As in Hassawi rice (n = 9; mean 43 ± 5 µg/kg). No significant difference between local and imported rice were noted for other toxic elements Cd, Pb and Hg (Mann–Whitney U test, 95% confidence limit). The data suggest that, in KSA, consumption of locally grown Saudi rice (Hassawi rice) rather than imported rice would lead to a lowering of i-As exposures. Although there were limited samples analysed in this study, the data showed that exposure to Pb, Cd or Hg from typical consumption in KSA of either local or imported rice is not predicted to lead to significant substantial carcinogenic risks. Similarly, exposure to i-As for a typical Saudi Arabian adult seems not to give rise to significant substantial carcinogenic risks. However, this study highlighted higher risks, especially with the increase in consumption of imported rice in the city of Najran, where exposure to i-As has reached the margin of safety (MOS) and EFSA BMDL_05_ values for skin cancer risks, suggesting that long-term exposure may result in adverse health effects. This study showed that people’s exposure depends largely on their dietary habits. Based on the experimental data and theoretical analysis presented in this work, the conclusion can be made that the consumption of locally grown Saudi rice (Hassawi rice) rather than imported rice can lead to a lowering of i-As exposures for males and females in KSA. Our findings indicate that it may be beneficial to increase local rice production, which is consistent with the targets of the Kingdom's Vision 2030, which is to raise domestic output and expand its global availability.

## Supplementary Information

Below is the link to the electronic supplementary material.

Supplementary file1 (DOCX 28 KB)

## Data Availability

Data is provided within the manuscript and/or supplementary information files.
