# Supplementary material for: Chemical analysis of toxic elements: total cadmium, lead, mercury, arsenic and inorganic arsenic in local and imported rice consumed in the Kingdom of Saudi Arabia
Source: Environ Geochem Health. 2024 Nov 16;46(12):518. doi: 10.1007/s10653-024-02280-0 (PMC11569018; doi:10.1007/s10653-024-02280-0)
Supplement: Supplementary file 1 — Supplementary file1 (DOCX 28 KB) [file 10653_2024_2280_MOESM1_ESM.docx]

**Supplementary material**

**Chemical analysis of total lead, cadmium, mercury, arsenic and inorganic arsenic in local and imported rice consumed in the Kingdom of Saudi Arabia**

May M. Alrashdi^ac^*, Abby Ragazzon-Smith^b^, Ilya Strashnov^a^ and David A. Polya^b^

*^a^Department of Chemistry, School of Natural Sciences, Faculty of Science and Engineering, The University of Manchester, Manchester, M13 9PL, UNITED KINGDOM*

*^b^Department of Earth and Environmental Sciences, School of Natural Sciences, Faculty of Science and Engineering, The University of Manchester, Manchester, M13 9PL, UNITED KINGDOM*

*^c^Department of Chemistry, College of Science, Jouf University, P.O. Box: 2014, Sakaka, Kingdom of Saudi Arabia*

*Corresponding author: [mmrashdi@ju.edu.sa](mailto:mmrashdi@ju.edu.sa); [david.polya@manchester.ac.uk](mailto:david.polya@manchester.ac.uk)

**Table S1.** Microwave digestion (CEM Mars (240/50)) parameters of rice samples for elemental (As, Cd, Hg & Pb) and hot block digestion system (Anton Paar) for As Speciation.

| **Total As, Cd, Hg & Pb**  **(Microwave digestion)** | 1600W power:100 % Ramp time 10 min 170°C Hold time: 20 min  Cooling time is 40-45 min. |
| --- | --- |
| **Arsenic speciation**  **(Hot block digestion system)** | 5 min ramp 95°C Hold for 90 min |

**Table S2.** Operating conditions of the Agilent 8900 ICP-MS/MS for As, Cd, and Pb analysis in KSA rice samples.

| **ICP-MS/MS Agilent 8900 conditions** | | **For As, Cd and Pb analysis** | |
| --- | --- | --- | --- |
|  |  |  |  |
| **Acquisition (ACQ) Mode** | | Spectrum | |
| **Peak Pattern** | | 1 point | |
| **Replicates** | | 4 | |
| **Stabilization Time** | | 35 sec | |
| **RF power** | | 1550 W | |
| **Nebulizer gas flow** | | 1.09 L/min | |
| **Nebulizer Pump** | | 0.10 rps | |
| **Sampling depth** | | 8.0 mm | |
| **Spray chamber temperature** | | 2 °C | |
| **MS/MS Mode** | **Integration Time/Mass** | As 75 -> 91  Rh 103 -> 103  Cd 111 -> 111  Pb 208 -> 208 | 3 sec  0.2 sec  3 sec  3 sec |
| **Rinse for As, Cd, and Pb measurement** | | Rinse Speed (Nebulizer Pump)  Rinse at Rinse Port (Sample)  Rinse at Rinse Port (Std) | 0.3 rps  10 sec |

**Table S3.** Operating conditions of the (i) Agilent 7700 ICP-MS and (ii) Agilent 1260 HPLC for Hg and Arsenic speciation analysis in KSA rice samples.

| **(i) ICP-MS** **Agilent 7700 conditions** | | **For As species analysis** | | **For Hg analysis** | | |
| --- | --- | --- | --- | --- | --- | --- |
|  | |  |  |  |  |  |
| **Acquisition (ACQ) Mode** | | Spectrum | | | | |
| **Peak Pattern** | | 1 point | | | | |
| **Replicates** | | 1 | | | 5 | |
| **Stabilization Time** | | 10 sec | | | | |
| **RF power** | | 1550 W | | | | |
| **Nebulizer gas flow** | | 1.00 L/min | | | | |
| **Nebulizer Pump** | | 0.30 rps | | | | |
| **Sampling depth** | | 4.0 mm | | | 8.0 mm | |
| **Spray chamber temperature** | | 2 °C | | | | |
| **MS/MS Mode** | **Integration Time/Mass** | As 75  Rh 103 | - 1. sec   0.01 sec | | Hg 201  Au 197  Rh 103 | 3 sec  0.1 sec  1 sec |
| **Rinse for Hg measurement** | | Rinse Speed (Nebulizer Pump)  Rinse at Rinse Port (Sample)  Rinse at Rinse Port (Std) | | | 0.5 rps  100 sec | |
|  |  | Rinse vial 1, 2 gold solution ((Au) 500 µg/L) and vial 3 nitric acid. | | | 0.2 rps  200 sec | |

| **(ii) HPLC Agilent 1260 conditions for As species analysis** | | |
| --- | --- | --- |
| **Injection volume** | 90 µL | |
| **Guard column** | Hamilton | |
| **Column** | Hamilton PRP-X 110S Anion Exchange | |
| **Dimensions / Particle size** | 100 x 2.1 mm, 7 µm | |
| **Mobile phase flow rate** | 1.500 mL/min | |
| **Mobile phase A (Eluent)** | 100 mmol ammonium carbonate in water with 2% Methanol (MeOH) (pH = 9.95 ± 0.05), (HCL) buffer solution MeOH | |
| **Mobile phase B (Eluent)** | Water with 2% MeOH | |
| **Mobile phase C (Eluent)** | Water with 2% MeOH | |
| **Mobile phase D (Eluent)** | Water with 2% MeOH | |
| **Gradient program** | 0 min.  6 min  7 min  9 min  9.50 min | 0.5%A, 50.0%B, 0.0%C, 49.5%D  22.0%A, 39.0%B, 0.0%C, 39.0%D  100.0%A, 0.0%B, 0.0%C, 0.0%D  100.0%A, 0.0%B, 0.0%C, 0.0%D  0.5%A, 50.0%B, 0.0%C, 49.5%D |
| **ACQ Mode** | Time resolved analysis (TRA) | |
| **Stop time mode** | Time set (10.00 min) | |

**Table S4.** Akaike information criterion (AIC) calculated for different distribution parameters where the AIC value is shown in bold this indicates the best fitted distributions.

|  | **AIC value for different fitted distributions** | | | | |
| --- | --- | --- | --- | --- | --- |
| **Element** | Normal | Lognormal | Weibull | Exponential | Gamma |
| i-As (Imported rice) | 6.755 | **6.497** | 6.955 | 8.716 | 6.558 |
| i-As (Local rice) | 7.386 | 7.329 | 7.640 | 12.605 | **7.232** |
| Cd (Imported rice) | 6.420 | **5.082** | 6.034 | 5.281 | 5.462 |
| Cd (Local rice) | 8.268 | 7.488 | 7.098 | 6.409 | **7.055** |
| Pb (Imported rice) | 6.173 | **4.609** | 5.311 | 4.655 | 5.234 |
| Hg (Imported rice) | 6.904 | **4.851** | 5.455 | 4.886 | 5.498 |
| Hg (Local rice) | 8.559 | **7.941** | 8.388 | 8.716 | 8.096 |
